# Therapeutic Notes

**Published:** 1907-12-14

**Authors:** 


### Therapeutic Notes.

### Geosote and Eosote.

These substances have been introduced as a remedy for phthisis and are identical chemically and physically—namely, isovalerianic acid united with methyl-pyrocatechin to form an "ester." This is a clear, oily fluid, fairly stable, with a strong aromatic odour, which is, however, not generally regarded as unpleasant. It is practically insoluble in water and only slightly soluble in alcohol, ether, and other usual solvents, including dilute acid and alkaline solutions. This compound has the advantage of being unirritating to the mucous membrane of the gastro-intestinal tract, and thus can be prescribed for long periods.

Geosote is administered either by the mouth or subcutaneously. It may also be employed as a local application. Its burning taste may be an objection to its use in children, or even in adults, so that for the latter it is best prescribed in gelatine capsules. Children, however, as a rule, find these difficult to swallow, and for them it may be prescribed in mucilage mixture, or in drops on a lump of sugar. The dose varies according to the object for which the drug is given. Five to fifteen minims three times a day may be given in chronic tuberculosis of the lungs for many months together; in typhoid or other conditions when an antiseptic action on the intestinal canal is required, two or three minims should be administered every hour, or more than a drachm in 24 hours. For tuberculous patients, however, subcutaneous injections are much recommended, as being the most efficacious method of administration. Schneider (Beitr. zur Klin. der Tuberk, Bd. v. p. 17) finds geosote of special advantage, owing to its purity, and the consequent absence of irritation and pain at the site of injection. Slight cases require seven minims every week injected into the flank or back; more serious cases will take fifteen minims. Full antiseptic precautions are, of course, necessary. A rise of temperature is an indication for stopping the injections, which should also be omitted during menstruation or in case of hæmoptysis. The remedy should be given in sufficient quantities to produce a smell of creosote in the stools. It should after prolonged use be recognisable in the urine two to five days after the last dose. It is often noticed in the breath, but is not usually perceived as a "taste" by the patient.

The local use of geosote consists of injections into tuberculous glands, which, unless caseation has occurred, have been observed to recede under this treatment (Risck). It has also been applied to lupus and injected into tuberculous sinuses. Geosote is prepared by Wendt of Berlin.

*Sirolin* is a combination of creosote or guaiacol, with a specially prepared extract of orange, which masks the taste, besides acting as a stimulant to the appetite owing to its bitter principles. The dose is half a teaspoonful for children up to a maximum of half an ounce for adults. It is said to have a beneficial effect on the course of pertussis.

*Sulfosote* is a similar preparation, but is much cheaper. It has a pleasant bitter-sweet taste, and is devoid of odour. It can be readily mixed with water, milk, alcohol, etc. The dose is the same as that of sirolin, and the indications are phthisis and other chronic lung affections for which creosote or its preparations are usually given.

*Thiocol* is a soluble creosote derivative, almost without odour or taste. Unlike its parent substance, it is not irritant, but is a stomachic, increasing the appetite and improving digestion and assimilation. It is absorbed up to 70 or 80 per cent., and is not split up into guaiacol in the stomach or intestines. It may be given even when hæmoptysis is taking place. The dose is 5 grains, in powder or tablets. A syrup is also prepared, and is said to be well tolerated by children.

These three creosote compounds are manufactured by Hermann La Roche and Co., of Bâle.

